# Reciprocal regulatory circuits coordinating EMT plasticity

**DOI:** 10.15698/cst2017.12.117

**Published:** 2017-12-01

**Authors:** Maren Diepenbruck, Gerhard Christofori

**Affiliations:** 1 Department of Biomedicine, University of Basel, 4058 Basel, Switzerland.

**Keywords:** EMT, invasion, miRNAs, metastasis, transcription factors

## Abstract

Epithelial to mesenchymal transition (EMT) as well as its reversal process, mesenchymal to epithelial transition (MET), are essential and well-controlled cellular processes during embryonic development. Tightly controlled regulatory mechanisms guide an EMT/MET plasticity and enable cells to switch forth and back between different cell morphologies and functional capabilities to endow the necessity of tissue plasticity. However, aberrant and uncontrolled activation of these processes during malignant tumor progression promotes primary tumor cell invasion, cancer cell dissemination and metastatic outgrowth. In a recent study (Nat Commun; doi: 10.1038/s41467-017-01197-w), we have reported on the post-transcriptional control of normal and cancer-associated EMT by miRNAs and identified a novel, critical double-negative feedback regulation of the thus far unknown miRNA miR1199 and the key EMT transcription factor Zeb1.

In the context of malignant tumor progression epithelial tumor cells can gain metastatic properties via an EMT induced by diverse extracellular stimuli (e.g. transforming growth factor β, TGFβ), which enables cancer cells to invade the surrounding tissue, disseminate through the blood circulation, seed distant metastases, and also escape therapy. Such major morphological and phenotypic changes require global adaptations of a cell`s transcriptomic landscape.

MiRNAs are a class of non-coding RNAs, which can alter gene expression during an EMT on the post-transcriptional level by either promoting mRNA degradation or preventing mRNA translation. In order to identify critical miRNAs functional during normal and cancer-associated EMT/MET plasticity, we first performed miRNA-sequencing on a detailed time course of a TGFβ-induced EMT in normal murine mammary gland cells. MiRNAs displaying a strong differential regulation in transcription were further tested for their functional contribution to an EMT and to mesenchymal tumor cell migration. We identified miR-1199-5p with reduced transcriptional expression and promoter activity during a TGFβ-induced EMT in various cellular models of EMT. Forced expression of miR-1199-5p in mouse and human untransformed mammary gland cells was sufficient to block EMT, decrease mesenchmyal breast cancer cell migration and invasion *in vitro* and, most importantly, to reduce the number of tumor cells circulating in the blood stream of tumor-bearing mice and the formation of lung metastasis *in vivo*. We further have reported that miR-1199-5p is embedded in a reciprocal regulation with the EMT transcription factor Zeb1. MiR-1199-5p represses the transcript levels of Zeb1 in epithelial cells on the post-transcriptional level. Upon the induction of an EMT, Zeb1 binds to a specific E-box motif in the promoter region of the miR-1199 gene and represses its promoter activity (**Figure 1**). Interestingly, the Zeb transcription factor family member Zeb2 also negatively controls the miR-1199 promoter activity to the same extent as Zeb1. However, no miR-1199-5p binding site was identified in the Zeb2 3` untranslated region (3`UTR) and we have not observed a regulation of Zeb2 mRNA by miR-1199-5p. In summary, we have reported the identification and characterization of a novel miRNA regulating an EMT by repressing the expression of the EMT transcription factor Zeb1.

**Figure 1:**
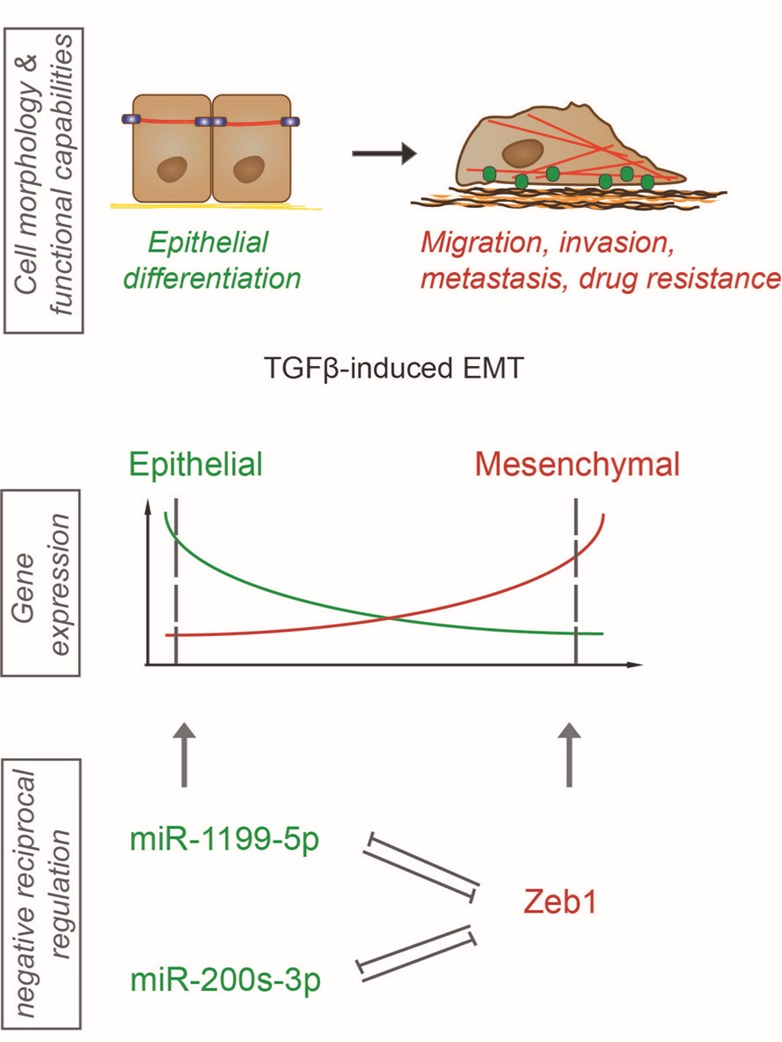
FIGURE 1: MiR-1199-5p and Zeb1 regulation and function during TGFβ-induced EMT. Schematic representation of the results reported by Diepenbruck et al. on the mechanistic function of miR-1199-5p during TGFβ-induced EMT in normal mammary cells and in breast cancer cells. Upon EMT induction, the transcript levels of miR-1199-5p (green) decrease while Zeb1 (red ) transcription increases. MiR-1199-5p as well as members of the miR-200 family and Zeb1 control each other on the transcriptional as well as on the post-transcriptional level in a negative reciprocal regulation. Forced expression of miR-1199-5p prevents TGFβ-induced EMT, mesenchymal tumor cell migration, invasion and metastasis formation.

Along with miR-1199-5p, we also identified in our EMT screen two well-known mediators of an epithelial cell phenotype, the miR-200 family members miR-200b-3p and miR-429-3p. The miR-200 family consists of five family members (miR-200a/b/c, miR-141, miR-429), which act as guardians of an epithelial morphology. They are highly expressed in epithelial cells, and their transcript levels decrease during an EMT. Interestingly, miR-200 family mem-bers are also embedded in a double-negative feedback regulation with Zeb1. Very comparable to what we observe with miR-1199-5p and Zeb1, miR-200s repress Zeb1 expression, while Zeb1 repress miR-200 expression. This notion begs the question whether miR-1199 and miR-200 family members are redundant in the regulation of Zeb1 expression and in the regulation of an EMT or whether they exert distinct activities. RNA-sequencing and differential gene expression analyses of cells overexpressing the various miRNAs revealed an overall stronger effect of miR-200b-3p and miR-429-3p in blocking an EMT than miR-1199-5p. One can only speculate whether this is due to a direct post-transcriptional repression of two other EMT master regulators, Snail1 and Zeb2, by miR-200b-3p and miR-429-3p. Further differential gene expression analyses revealed that miR-200b-3p, miR-429-3p and miR-1199 share only six target genes, among them Zeb1, while each of them has a larger number of individual target genes.

The gain of function approaches of miR-1199-5p and miR-200 family members clearly demonstrate that the transcriptional downregulation of these miRNAs is required for an EMT as well as mesenchymal tumor cell migration and invasion. In loss of function studies for all the individual miRNAs (miR-1199-5p, miR-200b/c-3p), using miRNA inhibitors and miRNA sponge constructs, we failed to observe an effect on Zeb1 mRNA expression levels and overall EMT induction. Since these miRNAs operate on the same transcription factor in a reciprocal manner, we suspect that the loss of one regulator is not enough to destabilize such a "buffered system".

Regulatory feedback loops consisting of a miRNA and key EMT transcription factors which control EMT and malignant tumor progression have now been reported by many independent research groups. Such reciprocal regulations are central molecular switches which provide functional robustness and enable a tight control of cell identity and regulate the extent of cellular plasticity, for example during an EMT.

